# High-Frequency Ultrasound Imaging to Evaluate Liver Fibrosis Progression in Rats and Yi Guan Jian Herbal Therapeutic Effects

**DOI:** 10.1155/2013/302325

**Published:** 2013-10-23

**Authors:** Wei Chen, Jiun-Yu Chen, Yu-Tang Tung, Hsiao-Ling Chen, Chia-Wen Kuo, Chia-Hui Chuang, Kowit-Yu Chong, Frank Chiahung Mao, Chuan-Mu Chen

**Affiliations:** ^1^Department of Life Sciences, Agricultural Biotechnology Center, iEGG Center, National Chung Hsing University, Kuo Kuang Road, Taichung 402, Taiwan; ^2^Division of Pulmonary and Critical Care Medicine, Chia-Yi Christian Hospital, Chia-Yi 600, Taiwan; ^3^Center for Nanomedicine Research, National Health Research Institutes, Miaoli 350, China; ^4^Department of Bioresources, Da-Yeh University, Changhwa 515, Taiwan; ^5^Taichung Armed Forces General Hospital, Taichung 411, Taiwan; ^6^Department of Medical Biotechnology and Laboratory Science, Chang Gung University, Tao-Yuan 333, Taiwan; ^7^Department of Veterinary Medicine, National Chung Hsing University, Taichung 402, Taiwan

## Abstract

The animals used in liver fibrosis studies must usually be sacrificed. Ultrasound has been demonstrated to have the ability to diagnose hepatic fibrosis and cirrhosis in experimental small-animal models. However, few studies have used high-frequency ultrasound (HFU, 40 MHz) to monitor changes in the rat liver and other hollow organs longitudinally. In this study, liver fibrosis was induced by administering dimethylnitrosamine (DMN) in SD rats, aged 8 weeks, for three consecutive days per week for up to 4 weeks. A Chinese herbal medicine Yi Guan Jian (YGJ) was orally administered (1.8 g/kg daily) to DMN-induced liver fibrosis rats for 2 weeks. Compared with the normal control rats, rats treated with DMN for either 2 weeks or 4 weeks had significantly lower body weights, liver indexes and elevation of hydroxyproline, GOT, and GPT contents. YGJ herbal treatment remarkably prevented rats from DMN-induced liver fibrosis. The HFU scoring results among the normal controls, 2-week DMN-treated rats, 4-week DMN-treated rats, and combined 2-week YGJ therapy with 4-week DMN-treated rats also reached statistical significance. Thus, HFU is an accurate tool for the longitudinal analysis of liver fibrosis progression in small-animal models, and the YGJ may be useful in reversing the development of hepatic fibrosis.

## 1. Introduction

Hepatic fibrosis has been reported to be a response to necrosis and inflammation caused by various factors such as infection, intoxication, and detrimental factors. Hepatic fibrosis leads to the activation of Kupffer cells, mononuclear cells, and hepatic stellate cells, resulting in the degeneration of the hepatic cords. The persistence of the insult disrupts the normal hepatic architecture, leading to the development of regenerative nodules and vascular dysfunction, which are used to determine the cirrhosis stage [1, 2]. Liver biopsy is the major tool for diagnosing hepatic fibrosis or cirrhosis and assessing the severity of an injured liver. However, this procedure has several drawbacks, including its invasiveness, the likelihood of sampling errors, and the potential for medical complications. 

Ultrasound has been demonstrated to have the ability to diagnose hepatic fibrosis and cirrhosis in experimental small-animal models [3–6]. However, the ultrasonic frequency used in these studies was always below 15 MHz, and the quality of the images was limited by the spatial resolution. High-frequency ultrasound (HFU) imaging, a recent developed technology, allows the imaging of small laboratory animals at a very high resolution [7, 8]. The frequency of such systems is above 20 MHz, and the spatial resolution is less than 100 *μ*m [8–10]. One of the most important advantages of this technology is the ability to image organs and tissue noninvasively in living animals. This ability allows disease progression in animal models to be monitored in longitudinal studies, which can shorten the observation time and reduce the number of sacrificed animals [11]. In our previous study, we identified the surgical anatomy of the rodent abdomen using high-frequency ultrasound (40 MHz) [12].

Traditional Chinese medicines (TCMs) are multi-ingredient extracts with low side effects in the treatment of chronic liver diseases [13, 14]. Yi Guan Jian (YGJ) decoction is a complex prescription of TCM consisting of 9 medical herbs [15, 16]. Although there is still no evidence-based clinical study, YGJ has been used to treat human liver fibrosis induced by hepatitis and has shown apparent efficacy in the reversal of liver fibrosis. The major active components of YGJ extract, ferulic acid, and catalpol, significantly inhibit the progression of hepatic fibrosis in carbon tetrachloride- (CCL4-) induced animal model study [17]. In addition, a more clinical representation of liver fibrosis model showed that the livers in dimethylnitrosamine- (DMN-) treated animals were shrunken with dark discoloration because of congestion [18]. However, the therapeutic effects of YGJ extract in the DMN-induced liver fibrogenesis have not been fully clarified.

In the present study, we used a commercially available high-frequency (40 MHz) ultrasound imaging system to detect and monitor the progression of hepatic fibrosis *in vivo* in the normal controls, 2-week DMN-treated rats, 4-week DMN-treated rats, and combined 2-week YGJ therapy with 4-week DMN-treated rats. The therapeutic effects of YGJ extract for liver fibrosis were extensively evaluated on histological examination, biochemical values, and hepatic fibrosis-related gene regulations.

## 2. Methods

### 2.1. Animal Model

Twenty male Sprague-Dawley (SD) rats (aged 8 weeks and weighing 250–300 g) were used. The animals were housed in an air-conditioned room at 23 ± 3°C with 55–60% relative humidity and a 12 h light/dark cycle. This study conformed to “The Guide for the Care and Use of Laboratory Animals” and was approved by the Ethics Committee of National Chung Hsing University (Approval no. 102-10). Two distinct models of experimental hepatic fibrosis were established in rats, one using DMN and one using normal saline (control group). In the DMN-induced fibrosis group, the rats were injected intraperitoneally with 1% DMN (10 mg/kg body weight) for three consecutive days per week for 2 weeks or 4 weeks as described previously [15, 19]. After treatment with DMN for 2 weeks, rats were orally administered 1.8 g/kg YGJ herbal extract (Koda Pharmaceutics Ltd., Taoyuan, Taiwan). The mock control group was given the same volume of phosphate-buffered saline (PBS) intraperitoneally for 4 weeks. The rats were sacrificed after 2 or 4 weeks of treatment with DMN or normal phosphate-buffered saline (PBS) or combined with YGJ herbal therapy.

### 2.2. High-Frequency Ultrasound (HFU) Examination

Animals were lightly anesthetized with 2.0–2.5% isoflurane vapor and placed in the supine position while breathing spontaneously. After the rats were anesthetized, the abdomen was shaved and further cleaned with a chemical hair remover to minimize ultrasound attenuation [19]. A commercially available HFU system (Visual Sonics Vevo 770, Toronto, Ontario, Canada) was used in this experiment. A transducer with a central frequency of 40 MHz, providing an axial resolution of 40 *μ*m with a 14.6 mm field of view, was used to image the rat's abdominal organs. When beginning to perform the HFU analysis, ultrasound gel was put on the skin as a coupling fluid. 

### 2.3. Longitudinal Evaluation of the Abdominal Organs by HFU

HFU was used to assess each rat on the first day of the 10th and 12th weeks of age. Each rat's abdomen was scanned carefully by three experienced researchers. The liver was examined to assess echogenicity, homogeneity, the surface of the margin, and intrahepatic vascular abnormalities. In addition, the size of the spleen and the presence or absence of ascites were also examined and recorded.

### 2.4. Ultrasound Scoring System

To evaluate the validity of HFU for the diagnosis of cirrhosis, we scored the ultrasonographic findings for the rats using a modified scoring system [20]. Items including the liver parenchyma echotexture, spleen size, ascites, vasculature, and the surface of the margin were used to score the severity of cirrhosis. The detailed scoring method is presented in Table 1.

### 2.5. Histological Examinations

To validate the *in vivo* imaging findings, rats were sacrificed, and their livers were excised immediately. To correlate the size and location of the previously observed lesions, visual inspection and *ex vivo* US of the liver were performed. The liver index (liver wet weight/body weight) and spleen index (spleen wet weight/body weight) were calculated. Liver tissue slices were fixed for 5 hours in Bouin's solution, followed by overnight exposure to 10% buffered formalin solution, pH 7.2, and then embedded in paraffin. Liver samples were sectioned (5 *μ*m) and stained with hematoxylin and eosin (H&E) and picrosirius according to standard protocols [21–23]. Masson's trichrome and Sirius Red staining were used to assess collagen deposition [15]. 

### 2.6. Tissue Hydroxyproline and Blood Biochemical Assays

Serum glutamate oxaloacetate transaminase (GOT) and glutamic pyruvic transaminase (GPT) concentrations were determined using a JSCC kit (Roche, Indianapolis, IN, USA). The hepatic hydroxyproline content was measured as described previously [15]. 

### 2.7. Detection of Fibrogenesis-Related mRNA by Quantitative RT-PCR

The mRNA expression of the fibrogenesis genes, alpha-smooth muscle actin (*α*-*SMA*) and collagen alpha-1(1) (*collagen* *α1-I*) was determined by semiquantitative RT-PCR. The housekeeping gene *glyceraldehyde-3-phosphate dehydrogenase* (*GAPDH*) was used as an internal control. Approximately 900 ng of total RNA extracted from liver tissue was reverse transcribed with MuLV Reverse Transcriptase using the GeneAmp RNA PCR Kit (Applied Biosystems, Foster, CA, USA) and oligo d(T)16 primers. Aliquots of the reverse transcriptase mix were used for PCR amplification of *α*-*SMA* (5′-TGGCTATTCCTTCGTGACTACTG-3′ and 5′-AAAGATGGCTGGAAGAGAGT CAC-3′), *collagen* *α1-I* (5′-GTTCGTGACCGTGACCTTGA-3′ and 5′-TTGGGGT TCGGGCTGATGTA-3′), and *GAPDH* (5′-ATCCCCAGAGCGTCATTCG-3′ and 5′-GAGAGAGCCCTGCCTGCC-3′). The transcription levels of each gene were normalized against those of GAPDH mRNA using a semiquantitative method as described previously [15, 24]. 

### 2.8. Statistical Analysis

The results were expressed as the mean ± standard deviation (SD). The differences between the DMN-treated group and the control group were evaluated using Student's *t*-test. Statistical significance was set at *P* < 0.05 (* or ^#^).

## 3. Results 

### 3.1. Correlation of Gross Liver Characteristics and HFU Image Findings **In Vivo ** in Control and DMN-Treated Rats

**Figure 1:**
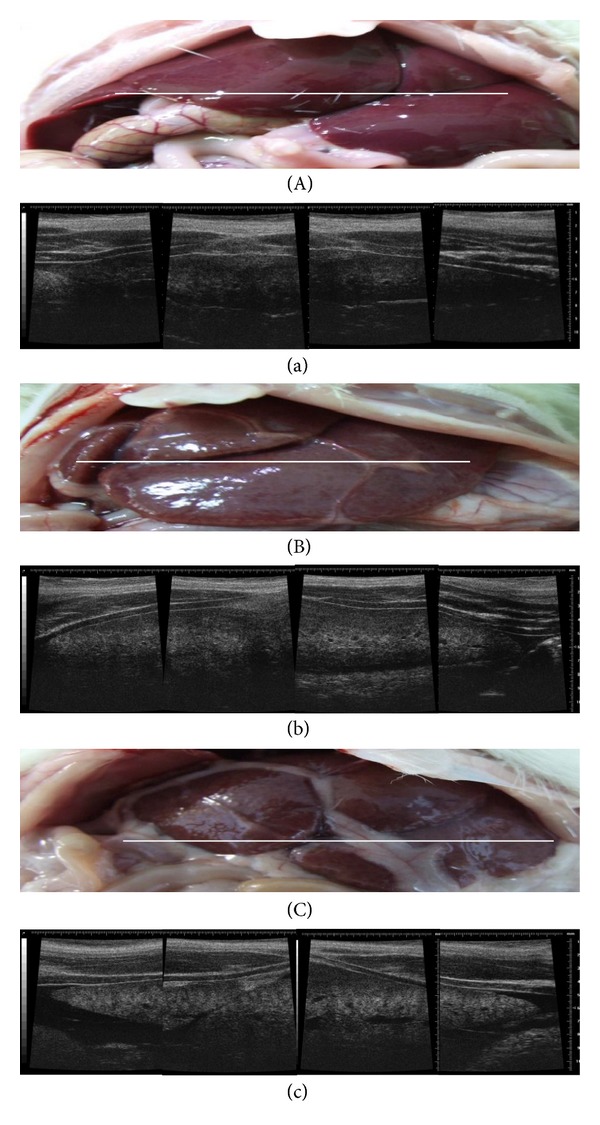
The gross appearance of a normal liver (A), 2-week DMN-treated liver (B), and 4-week DMN-treated liver (C). Panels (a), (b), and (c) are the ultrasonographic images (40 MHz) corresponding to panels (A), (B), and (C), respectively. The echogenicity of the DMN-treated rat livers was more hyperechoic than that of normal rat livers.

In Figure 1, panel (A) presents the gross appearance of a normal liver, panel (B) presents the gross appearance of the 2-week DMN-treated rat liver, and panel C presents the gross appearance of 4-week DMN-treated rat liver. Panels (a), (b), and (c) show the ultrasonographic images corresponding to the photographs in panels (A), (B), and (C), respectively. In the normal rats (Figure 1(A)), the liver was a soft, pinkish-brown organ consisting of major four lobes (the median lobe, right lobe, left lobe, and caudate lobe). In the 2-week DMN-treated rats, the liver's surface was relatively darker than that in the normal rats, and the margin of the liver was coated with a yellowish material (Figure 1(B)). In the 4-week DMN-treated rats, the liver was smaller and relatively dark brown and was coated with yellowish material along the fissures and margins (Figure 1(C)). In the ultrasonographic images, the normal liver parenchyma had a uniform, sponge-like texture with low echogenicity (Figure 1(a)). Passing through the parenchyma were blood vessels, which were observed as branching tubular structures that could be traced toward the portal or hepatic vein. In the 2-week DMN-treated fibrotic rats, the ultrasonographic images of the liver were relatively hyperechoic (Figure 1(b)). The most hyperechoic pattern was observed for the 4-week DMN-treated rat livers (Figure 1(c)). In addition, ascites was also detected by ultrasound in the 4-week DMN-treated rat livers (Figure 1(c)). 

### 3.2. Pathological Features in Normal Control and DMN-Treated Rats

**Figure 2:**
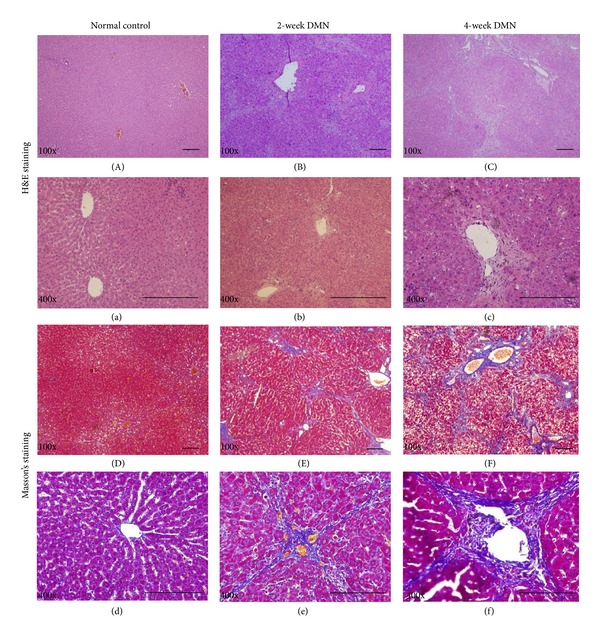
The normal lobular architecture with central veins and radiating hepatic cords was observed in normal control rats ((A), (a), (D), and (d)). Fatty degeneration, necrosis, the infiltration of inflammatory cells, and the apparent formation of fibrotic septa were present in the 2-week DMN-treated group ((B), (b), (E), and (e)) and 4-week DMN-treated group ((C), (c), (F), and (f)). Tissue stained with H&E and imaged at 100x ((A), (B), and (C)) or 400x ((a), (b), and (c)) magnifications. Tissue stained with Masson's trichrome and imaged at 100x ((D), (E), and (F)) or 400x ((d), (e), and (f)) magnifications.

To assess the reliability of HFU imaging, we performed hematoxylin and eosin (H&E) staining and Masson's trichrome staining to examine pathological alterations and collagen deposition (Figure 2). The normal lobular architecture with central veins and radiating hepatic cords was observed in the livers of normal control rats (Figures 2(A), 2(a), 2(D), and 2(d)). Fatty degeneration, necrosis, the infiltration of inflammatory cells, and the apparent formation of fibrotic septa were present in the 2-week DMN-treated group (Figures 2(B), 2(b), 2(E), and 2(e)) and became more severer in the 4-week DMN-treated group (Figures 2(C), 2(c), 2(F), and 2(f)). 

### 3.3. HFU Analysis of Associated Organs

**Figure 3:**
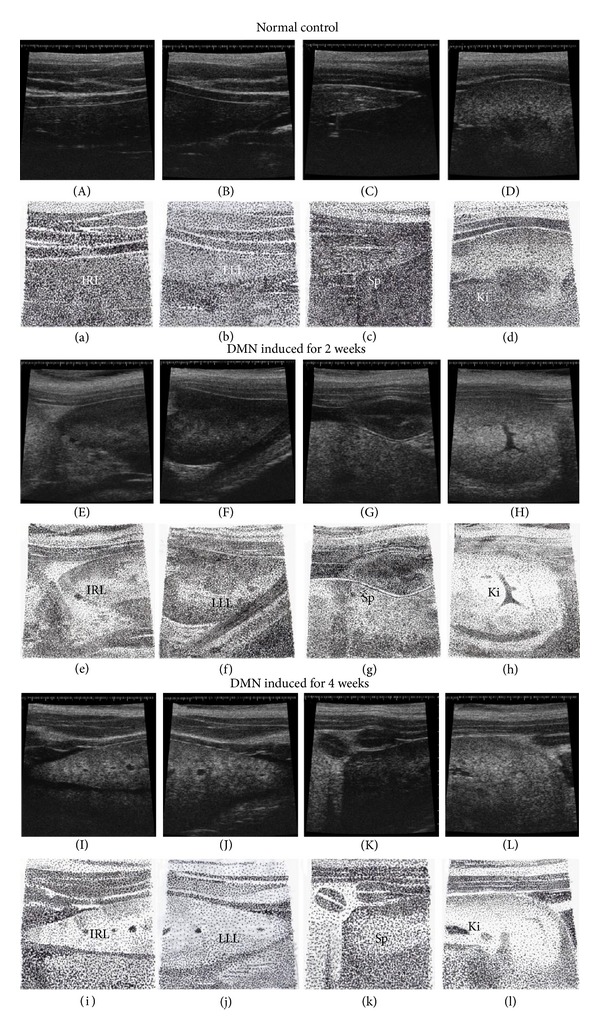
Ultrasonographic images (40 MHz) of normal control rats, 2-week DMN-treated rats, and 4-week DMN-treated rats. ((A), (B), (C), and (D)) The HFU images of the normal rat inferior right liver (IRL), lateral left liver (LLL), spleen (Sp), and left kidney (Ki), respectively. Panels (a), (b), (c), and (d) are the schematic diagrams of panels (A), (B), (C), and (D), respectively. ((E), (F), (G), and (H)) The 2-week DMN-treated rat inferior right liver, lateral left liver, spleen, and left kidney, respectively. Panels (e), (f), (g), and (h) are the schematic diagrams of panels (E), (F), (G), and (H), respectively. ((I), (J), (K), and (L)) The 4-week DMN-treated rat inferior right liver, lateral left liver, spleen, and left kidney, respectively. Panels (i), (j), (k), and (l) are the schematic diagrams of panels (I), (J), (K), and (L), respectively.

**Figure 4:**
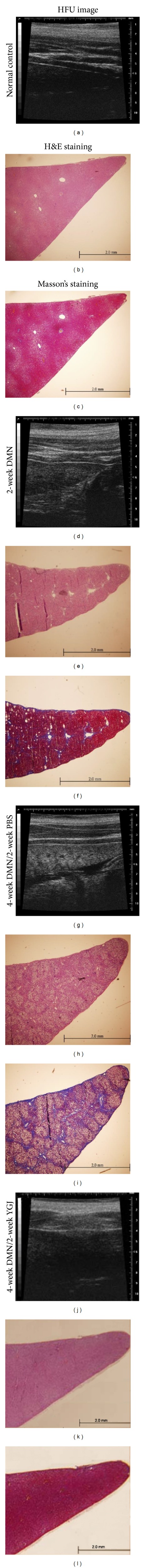
Pathological features for effects of the YGJ herbal therapy on DMN-induced liver fibrosis. ((a), (b), and (c)) The normal lateral left liver architecture with central veins and radiating hepatic cords was observed in normal control rats. ((d), (e), and (f)) Fatty degeneration, necrosis, infiltration of inflammatory cells, and apparent formation of fibrotic septa were present in the 2-week DMN-treated group. ((g), (h), and (i)) The more severer fibrotic liver architecture images were present in the 4-week DMN-treated group. ((j), (k), and (l)) The degree of liver fibrosis was significantly reduced in the YGJ-treated group. ((a), (d), (g), and (j)) Lateral left liver architectures of HFU (40 MHz) images. ((b), (e), (h), and (k)) Tissues stained with H&E, bar = 2.0 mm. ((c), (f), (i), and (l)) Tissues stained with Masson's trichrome, bar = 2.0 mm.

The livers of either 2-week (Figures 3(E), 3(e), 3(F), and 3(f)) or 4-week (Figures 3(I), 3(i), 3(J), and 3(j)) DMN-treated rats were relatively smaller than those of the normal rats (Figures 3(A), 3(a), 3(B), and 3(b)). The ultrasonographic images of the spleens of DMN-treated rats showed increased echogenicity (Figures 3(G), 3(g), 3(K), and 3(k)). The kidneys were also hyperechogenic in DMN-treated rats (Figures 3(H), 3(h), 3(L), and 3(l)). Ascites was frequently detected in the DMN-treated rats.

### 3.4. Ultrasound Scoring to Evaluate YGJ Therapeutic Effect

In the modified scoring system, the normal control rats were scored as 0. After 2 weeks of treatment with DMN, the average score was 1.6 ± 0.9, and after 4 weeks of DMN treatment, it was increased to 6.2 ± 0.8. As shown in Table 2, we can see that the ultrasound score was significantly different in the 2-week DMN-treated rats (*P* < 0.05) and the 4-week DMN-treated rats (*P* < 0.01) compared to normal control rats. The average score of the 4-week DMN-treated and 2-week YGJ therapy group was significantly decreased (2.8 ± 0.8, *P* < 0.05) compared to the 4-week DMN-treated rats without YGJ therapy. To evaluate interobserver and intraobserver variability in the HFU imaging assessment of the severity of fibrotic liver, three experienced operators independently graded the hepatic images as normal, fibrosis, or cirrhosis. Weighted kappa statistics were used to analyze interobserver and intraobserver agreement as shown in Tables S1 and S2 in Supplementary Material available online at http://dx.doi.org/10.1155/2013/302325. Results showed that the intraobserver agreement was 70–100% (*κ* = 0.53–1.0) and interobserver agreement was even higher from 93% to 100% (*κ* = 0.85–1.0). Therefore, this is a reliable sonographic assessment method for rat liver fibrosis and cirrhosis.

### 3.5. Physical and Biochemical Characteristics of the Normal, DMN-Treated, and DMN-Treated Combined YGJ Therapy Rats

As shown in Table 3, rats treated with DMN for either 2 weeks or 4 weeks were significantly different from the control rats in the terms of body weight, spleen index, hydroxyproline level, GOT level, and GPT level. However, rats treated with DMN and combined with 2 weeks YGJ therapy significantly decreased serum GOT and GPT levels and restored body weight compared to the DMN-treated without YGJ therapy group (*P* < 0.05). Moreover, the hepatic hydroxyproline concentration in the 4-week DMN-treated group increased 5.6-fold over the normal control group (*P* < 0.001) but significantly decreased in the YGJ therapeutic group (*P* < 0.01) compared to the DMN-treated alone group.

### 3.6. Effects of YGJ Herbal Therapy on Liver Histological Changes

Liver histopathological analyses consisted of tissue section staining with H&E and Masson's trichrome dye and combined with HFU imaging analysis as shown in Figure 4. Liver tissue from 4-week DMN-treated rats had more steatosis, fibrotic septa, necrotic hepatocytes, and incorporation of degenerated hepatocytes into pseudolobules as well as associated higher collagen content (Figures 4(g), 4(h), and 4(i)) than that of normal control rats (Figures 4(a), 4(b), and 4(c)) and 2-week DMN-treated rats (Figures 4(d), 4(e), and 4(f)). However, 4-week DMN-treated rats combined with YGJ herbal therapy for 2 weeks markedly alleviated the degree of liver fibrosis and significantly reduced the collagen deposition (Figures 4(j), 4(k), and 4(l)).

### 3.7. Effects of YGJ on Gene Expression of *α*-*SMA* and *Collagenα* **1-I**

**Figure 5:**
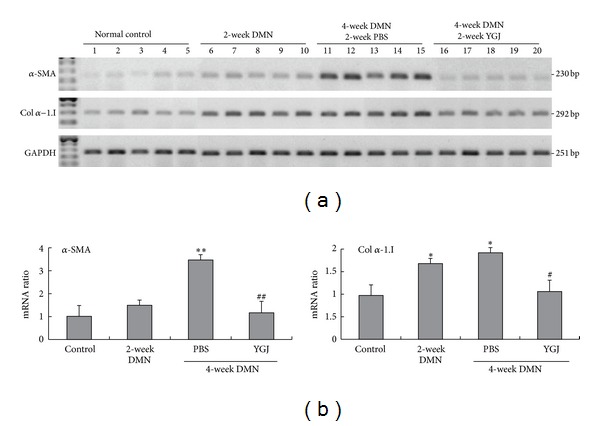
Fibrotic gene expression levels of *α*-*SMA* and Col *α*-*1.I* in normal control rats, DMN-induced fibrosis rats, and YGJ herb-treated rats. (a) Gene expression patterns were amplified by semiquantitative RT-PCR in four different groups including the normal control rats, 2-week DMN-treated rats, 4-week DMN-treated with 2-week PBS rats, and 4-week DMN-treated with 2-week YGJ rats (*n* = 5). (b) The expression levels of *α-SMA* and Col *α-1.I* were quantified using densitometry. Values were normalized against those for *GAPDH* mRNA expression as an internal calculation control. *P < 0.05; **P < 0.01 compared with the normal control group. ^#^ *P* < 0.05; ^##^ *P* < 0.01 compared with 4-week DMN-treated group.

Gene expression for *α-SMA* and *collagen* *α1-I*, which represent hepatic fibrosis factors, was analyzed using RT-PCR (Figure 5(a)). The mRNA levels of *α-SMA* and *collagen* *α1-I* were increased in either 2-week DMN-treated rats (1.5- and 1.7-fold) or 4-week (3.6- and 1.9-fold) DMN-treated rats, respectively, compared with the normal control group (*P* < 0.05). The YGJ herbal treatment significantly reduced the mRNA levels of *α-SMA* and *collagen* *α1-I* by 67% (*P* < 0.01) and 45% (*P* < 0.05), respectively compared with the 4-week DMN-treated alone group (Figure 5(b)).

## 4. Discussion 

In the current study, we demonstrated that high-frequency ultrasound (HFU, 40 MHz) imaging is a useful tool for the *in vivo* analysis of liver tissue during chronic toxin exposure and the induction of liver fibrosis in rats. In addition, we developed an ultrasound scoring system to help assess the severity of liver fibrosis in rats. The mean HFU imaging score increased with increasing fibrosis stage. It is a useful tool to evaluate the therapeutic efficacy of the YGJ herbal medicine treatment.

Small animals have been used as models for the investigation of toxin- or virus-induced liver injury for decades [25–28]. To confirm the pathological findings of liver damage, the majority of studies have sacrificed animals in the experimental and control groups at different time points during the study. This type of study design has two drawbacks. First, it lacks continuity. For example, mouse A is sacrificed after 2 weeks of toxin exposure, and mouse B is sacrificed after four weeks of toxin exposure. In this situation, we usually regard the pathological changes exhibited by mouse B as the continuation of the changes observed in mouse A. However, even if the animals belong to the same strain, there may be some bias. Second, this type of study design requires many more animal sacrifices. If a study does not use imaging to assess animals, animals must be sacrificed at every observational and interventional time point. 

Ultrasound has been used to analyze the livers of small animals for a long history [29, 30]. Initially, researchers found that diffuse liver disease appears ultrasonographically as a change in the liver's echogenicity relative to that of the renal cortex or spleen. To precisely assess the severity of liver disease, some quantitative methods and tools have been developed. Matsuhashi and colleagues found that the speed of the sound is useful for diagnosing fatty liver and cirrhosis [6]. Guimond et al. confirmed that quantitative ultrasonic tissue characterization can be used as a tool to continuously monitor chronic liver remodeling in mice [3]. Wang et al. suggested that stiffness measurements using acoustic radiation force can provide a quantitative assessment of the extent of fibrosis in the liver and can potentially be used for the diagnosis, management, and study of liver fibrosis [31]. Ho et al. suggested that ultrasound Nakagami imaging is a functional imaging tool to complement the use of conventional B-scanning in animal studies of liver fibrosis [32]. However, all studies mentioned above require the use of complicated or specific techniques or instruments. Due to technical advances, HFU, which refers to frequencies above 20 MHz, has become more readily available [7–10]. Fernández-Domínguez et al. reported that diagnostic HFU is an effective method for monitoring the progression of liver disease from steatosis to hepatocellular carcinoma in mice [33]. Similar to the results of their study, we found that HFU can be used as a noninvasive tool to monitor liver disease progression in rats.

One study showed that both low-frequency (2–5 MHz) and high-frequency probes (5–12 MHz) were reliable and effective alternatives to the histological staging of chronic liver diseases [34]. However, our study using a 40 MHz probe showed that the images had a higher resolution than those obtained with 20 MHz probes and that it was easier to monitor the detailed changes in disease progression using the 40 MHz probe. Another advantage of the 40 MHz probe is that the rat's spleen can be easily detected. When using a 20 MHz probe, the acoustic waves are hampered by the rat's ribs. 

The use of an ultrasound scoring system is a reliable method for evaluating the severity of diffuse liver disease in humans and small animals [5, 35, 36]. Yan et al. reported that an ultrasound scoring system, especially when combined with computed tomography (CT) and serum fibrosis markers, had a higher value in the noninvasive quantitative diagnosis of hepatic fibrosis in longitudinal studies [5]. In our study, the scores were significantly different between the normal control, 2-week DMN-treated, and 4-week DMN-treated rats. To reduce the bias of the interpretation of the ultrasound images, operators have to practice using the HFU system for at least 3 weeks before initiating a study. In addition, ultrasound images were recorded and analyzed by two operators working based on consensus.

According to Chinese medicinal theory, liver disease is caused by an epidemic pathogen and dampness heat and would result in stasis of the blood [37]. When liver disease progresses to liver cirrhosis, the result is a blood deficiency. Serum activity of GOT and GPT are the most commonly used biochemical markers of liver injuries [38]. The present examination of the progress of liver injury induced by a repeated administration of DMN found that both serum GOT and GPT activities markedly increased. The YGJ herbal therapy clearly repressed DMN-induced GOT and GPT activities, indicating that the YGJ extract has potent hepatoprotective effects. Hepatic hydroxyproline content, *α*-*SMA*, and collagen  *α*-*1.I* gene expression measurements also demonstrated that YGJ extract treatment significantly decreased the accumulation of collagen compared with the DMN-treated and PBS-administrated groups. The pathological changes of liver tissue obviously improved with YGJ extract treatment, indicating the inhibitory effect of the YGJ extract on hepatic fibrosis.

## 5. Conclusions 

It is evident that HFU provides higher-resolution images for monitoring the progression of liver disease in rats. The use of HFU may reduce the number of invasive procedures required and reduces the number of sacrificed animals. In addition, the ultrasound scoring system based on HFU is useful for detecting, grading, and monitoring the progression of liver disease in rats, and the results of this system correlate well with pathological findings. We also demonstrated that the primary mechanism of YGJ therapeutic effect could be protection against hepatic injury via reduced serum levels of GOT and GPT as well as reduction of the activated HSCs via downregulation of *α*-*SMA* and collagen  *α*-*1.I* mRNA expressions. It provides scientific evidence for the clinical use of YGJ extract in treating liver fibrosis and cirrhosis.

## Supplementary Material

Supplementary Table: It shows intraobserver agreement for severity of liver fibrosis and cirrhosis in different scoring parameters. It shows the relation between observer, parenchymal echotexture, ascites, and vasculature.

## Figures and Tables

**Table 1:** Scoring system for high-frequency ultrasound (HFU) signs for the assessment of liver fibrosis.

Characteristic	US sign	Score
Parenchymal echotexture^a^	Homogeneous echotexture	0
	Heterogeneous echotexture	1
	Coarse echotexture	2

Spleen size^b^	Normal	0
	Enlarged	1

Ascites^c^	Absence	0
	Presence	1

Vasculature^d^	Normal	0
	Obscure with normal diameter	1
	Stretched or broad vessels	2

Surface or margin^e^	Smooth	0
	Irregular	1

The cirrhosis score was determined as follows.

^a^Liver parenchyma (hepatic parenchyma away from major vessel or bile duct were used in evaluation): 0 score for homogenous appearance of the parenchyma; 1 score for heterogenous with finely scattered hypoechoic and hyperechoic areas; 2 score for coarse liver with irregular or patchy pattern.

^b^Spleen size index (calculated as product of oblique and diagonal diameter): 0 score for spleen size index <1.7 cm^2^; 1 score for larger spleen.

^c^Ascites: 0 score for absence of ascites; 1 score for presence of ascites.

^d^Vasculature: 0 score for smooth vessel wall; 1 score for obscured or blurred vessel with normal diameter; 2 score for irregular and narrowed vessel.

^e^Surface of margin: 0 score for smooth surface; 1 score for uneven surface or shallow, irregular nodular surface.

**Table 2:** Ultrasound characteristics (40 MHz) of fibrosis in normal liver, 2-week DMN-treated fibrotic liver, and 4-week DMN-treated fibrotic liver.

Treatment	Parenchymal echotexture	Spleen size	Ascites	Surface or margin	Vasculature	Sum	Average^†^
Normal control	0	0	0	0	0	0	
	0	0	0	0	0	0	
	0	0	0	0	0	0	0
	0	0	0	0	0	0	
	0	0	0	0	0	0	

DMN-induced fibrosis (2 wk)	1	0	0	0	0	1	
	0	0	0	0	1	1	
	1	0	0	0	0	1	1.6 ± 0.9*
	1	0	1	0	0	2	
	1	1	0	0	1	3	

DMN-induced fibrosis (4 wk)/PBS (2 wk)	2	1	1	1	2	7	
	2	1	1	0	2	6	
	2	1	0	1	2	6	6.2 ± 0.8**
	2	1	1	1	2	7	
	1	1	0	1	2	5	

DMN-induced fibrosis (4 wk)/YGJ (2 wk)	0	0	1	1	1	3	
	0	0	1	0	1	2	
	1	1	0	1	1	4	2.8 ± 0.8^∗,#^
	0	1	1	0	0	2	
	1	1	0	0	1	3	

^†^Each value represents the mean ± SEM of five rats.

*P < 0.05, **P < 0.01 versus normal control.

^#^ *P* < 0.05 versus DMN-induced fibrosis/PBS group.

**Table 3:** Body weight, liver index, spleen index, hydroxyproline level, GOT level and GTP level in the normal control group, 2-week DMN-treated group, and 4-week DMN-treated group.

Treatment^†^	Normal control	DMN-induced fibrosis (2 wk)	DMN-induced fibrosis (4 wk)/PBS (2 wk)	DMN-induced fibrosis (4 wk)/YGJ (2 wk)
Body weight (g)	471.3 ± 42.9	411.3 ± 34.7*	341.7 ± 40.4**	418.7 ± 36.9^∗,#^
Liver index (%)	4.2 ± 0.3	4.1 ± 0.4	3.5 ± 0.7**	4.3 ± 0.8^##^
Spleen index (%)	0.2 ± 0.0	0.5 ± 0.1**	0.4 ± 0.1*	0.4 ± 0.1*
Hydroxyproline (*µ*g/g liver)	208.7 ± 19.5	452.2 ± 67.8**	1187.1 ± 220.1***	403.5 ± 61.3^∗,##^
GOT (U/L)	57.2 ± 1.7	175 ± 22.4***	136.8 ± 6.4**	71.9 ± 8.3^∗,#^
GPT (U/L)	39.0 ± 7.1	83.4 ± 11.0**	97.8 ± 10.5**	52.0 ± 7.7^∗,#^

^†^Each value represents the mean ± SEM of five rats.

*P < 0.05, **P < 0.01, ***P < 0.001 versus normal control.

^#^ *P* < 0.05, ^##^ *P* < 0.01 versus DMN-induced fibrosis/PBS group.
