# Supplementary material for: High-Frequency Ultrasound Imaging to Evaluate Liver Fibrosis Progression in Rats and Yi Guan Jian Herbal Therapeutic Effects
Source: Evid Based Complement Alternat Med. 2013 Oct 23;2013:302325. doi: 10.1155/2013/302325 (PMC3819923; doi:10.1155/2013/302325)
Supplement: Supplementary file 1 — Supplementary Table: It shows intraobserver agreement for severity of liver fibrosis and cirrhosis in different scoring parameters. It shows the relation between observer, parenchymal echotexture, ascites, and vasculature. [file 302325.f1.docx]

**Supplementary Tables**

| **Table S1. Intraobserver agreement for severity of liver fibrosis and cirrhosis in different scoring parameters** | | | | | | |
| --- | --- | --- | --- | --- | --- | --- |
| Observer | Parenchymal echotexture | | Ascites | | Vasculature | |
|  | κ | Agreement (%) | κ | Agreement (%) | κ | Agreement (%) |
| A | 1 | 100 | 0.71 | 82 | 1 | 100 |
| B | 1 | 100 | 0.53 | 70 | 1 | 100 |
| C | 1 | 100 | 0.85 | 93 | 1 | 100 |

Kappa value (k) for the strength of agreement: k<0.00: poor; k=0.00-0.20: slight; k=0.21-0.40: fair; k=0.41-0.60: moderate; k=0.61-0.80: substantial; k=0.81-1.00: almost perfect.

| **Table S2. Interobserver agreement for severity of liver fibrosis and cirrhosis in different scoring parameters** | | | | | | |
| --- | --- | --- | --- | --- | --- | --- |
| Observer | Parenchymal echotexture | | Ascites | | Vasculature | |
|  | κ | Agreement (%) | κ | Agreement (%) | κ | Agreement (%) |
| A and B | 1 | 100 | 0.86 | 93 | 1 | 100 |
| A and C | 1 | 100 | 0.85 | 93 | 1 | 100 |
| B and C | 1 | 100 | 0.85 | 93 | 1 | 100 |
